# Association of triglyceride‑glucose index in branch retinal vein occlusion

**DOI:** 10.1007/s00417-024-06376-2

**Published:** 2024-02-01

**Authors:** Hatice Aslan Sirakaya, Ender Sirakaya

**Affiliations:** 1 Department of Internal Medicine, Health Science University, The Kayseri City Hospital, Kayseri, Turkey; 2 Department of Ophthalmology, Health Science University, The Kayseri City Hospital, Kayseri, Turkey

**Keywords:** Branch retinal vein occlusion, BRVO, Triglyceride-glucose index, TyG index, Atherosclerosis

## Abstract

**Background:**

To investigate the association between the triglyceride-glucose (TyG) index and newly diagnosed branch retinal vein occlusion (BRVO) in patients.

**Methods:**

The study included 57 individuals with BRVO and a control group comprising 50 healthy volunteers matched for age and gender. Detailed eye examinations were conducted, and various blood biochemistry and hematological parameters were recorded. The TyG index was calculated using fasting plasma glucose and triglyceride values.

**Results:**

The mean age was 61.4 ± 9.6 years for the BRVO group and 60.6 ± 10.3 years for the control group. The TyG values were significantly higher in the BRVO group when compared to the control group (8.84 ± 0.41 vs. 8.52 ± 0.29, *p* < 0.001). Multivariate analysis revealed that the TyG index independently predicted BRVO (odds ratio = 2.58, 95% confidence interval = 1.69–3.93; *p* < 0.001). In receiver operating characteristics analysis, the TyG index had an area under the curve of 0.749, and a TyG index higher than 8.52 predicted BRVO with 83% sensitivity and 70% specificity.

**Conclusions:**

This study establishes a significant association between an elevated TyG index and BRVO. Consequently, the TyG index could serve as a valuable predictive tool for identifying individuals at risk for BRVO.

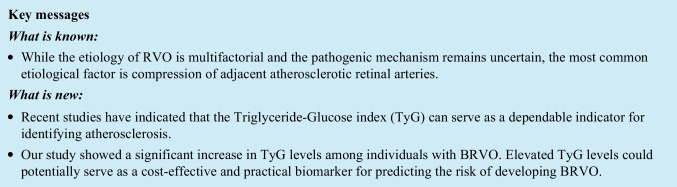

## Introduction

Retinal vein occlusion (RVO) leads to notable vision loss and ranks as the second most common vascular ailment affecting the retina. RVO can be classified into two subtypes depending on the site of the obstructed blood vessel: branch retinal vein occlusion (BRVO) and central retinal vein occlusion (CRVO). BRVO, with an annual incidence of 2.14/1000 in the population over 40 years of age, exhibits a higher prevalence compared to CRVO [1, 2].

There is not yet a full understanding of the cause of BRVO. The primary issue lies in thrombosis and the rise in intravascular pressure due to the obstruction of the vein, leading to the leakage of blood cells and plasma into the retina. Numerous research findings have validated the systemic risk factors associated with the development of BRVO. These factors encompass advancing age, hypertension, diabetes mellitus [3], dyslipidemia [4], cardiovascular disease [5], hypercoagulability [6], oral contraceptives, pregnancy, and ocular factors such as intraocular hypertension or glaucoma [7]. In the pathogenesis, arteries and veins, which share a common adventitial sheath, are thought to provide an environment for the development of BRVO and narrowing of the venous lumen in the arteriovenous transition zones. The formation of arteriolar sclerosis might augment the risk of occlusion in this area by increasing the stiffness of the crossed artery. The atherosclerotic retinal artery leads to turbulence in the blood flow, which can damage the endothelial cells and induce clot formation [8]. Extensive research from large-scale studies indicates that individuals with systemic arteriosclerotic vascular disease are at a heightened risk for this condition [9].

In recent years, it has been reported that the triglyceride-glucose (TyG) index calculated as ln(Logaritma Naturel)[fasting triglycerides (mg/dL) × fasting glucose (mg/dL)/2] is a simple and reliable marker for the determination of insulin resistance and atherosclerosis. A robust association exists between the TyG index and atherosclerosis-related diseases, including myocardial infarction and ischemic stroke [10]. It has been shown that the high TyG index is associated with high arterial stiffness and the TyG index has clinical significance in the evaluation of microvascular damage.

According to the literature review, the role of the TyG index in BRVO has not yet been discussed. The TyG index can be a new useful indicator in the development of BRVO. This study aimed to investigate the TyG index in patients with BRVO.

## Methods

This single-center, cross-sectional study was conducted by the Department of Ophthalmology at Science University, The Kayseri City Hospital, Kayseri, Turkey. The research adhered to the principles of the Declaration of Helsinki, and it received approval from the institutional review board and ethics committee. Prior to commencing the study, all participants willingly consented to take part, providing both verbal and written informed consent. In the study, 57 patients with newly diagnosed acute stage of BRVO (group I) were compared with 50 healthy controls (group II). The control group consisted of healthy individuals who were scheduled for eyelid or cataract surgery. Participants of the same age and gender were included in both the study and control groups.

Every participant underwent a comprehensive eye examination, which included the use of Snellen charts to measure best-corrected visual acuity, slit-lamp biomicroscopy, Goldmann applanation tonometry to measure intraocular pressure, and a dilated stereoscopic fundus examination. Fundus fluorescein angiography (FFA) (FFA, VISUCAM NM/FA; Carl Zeiss Meditec AG, Dublin, CA, USA) and spectral domain optical coherence tomography (Heidelberg Engineering, Heidelberg, Germany) were used to examine the retina. An experienced retinal specialist (ES) diagnosed BRVO with dilated fundus examination, OCT, and/or FA, determining on the location of the retinal vascular obstruction. The diagnosis of BRVO was made by the detection of scattered superficial and deep retinal hemorrhages, arteriovenous notching, dilatation due to obstruction distal to the arteriovenous crossing region, narrowing due to decreased blood flow in the proximal part, cotton spots, hard exudates, retinal edema in the arteriovenous passage corresponding to the occluded vessel in dilated fundus examination. Vasculitis-related BRVO was ruled out due to the absence of perivascular sheath and vitritis.

Participants were excluded if they had a history of uncontrolled hypertension and diabetes mellitus, serious cardiovascular disease, stroke, renal failure, hepatic disorders, abnormal thyroid function, chronic obstructive pulmonary disease, anemia, malignancy, acute infectious disease, chronic systemic inflammatory disease, or if they were active smokers or had a history of excessive alcohol consumption. Participants were excluded if they had a history of serious ocular diseases such as uveitis, scleritis, or retinal disease (with the exception of BRVO).

Each blood sample was obtained from all patients following a fasting period of 8–12 h. The following measurements were recorded: fasting blood glucose (FBG), total cholesterol (TC), high-density lipoprotein (HDL), low-density lipoprotein (LDL), triglyceride (TG), Hemoglobin A1c (HbA1c), and high-sensitivity C-reactive protein (hsCRP). The triglyceride-glucose index (TyG) was computed using the formula Natural Logarithm [fasting triglycerides (mg/dL) × fasting plasma glucose (mg/dL)/2] [11].

The statistical analysis was conducted using IBM Statistical Package for the Social Sciences version 25 (IBM Corp., Armonk, NY, USA). Data normality was assessed using the Shapiro–Wilk test. Continuous data were presented as mean ± standard deviation (SD), while categorical variables were expressed as numbers and percentages. Categorical data were assessed through the Chi-square test, and the comparison of variables between the two groups was performed using an independent sample test. Logistic regression analyses were carried out to identify independent predictors of TyG. Receiver operating characteristics (ROC) curve analysis was employed to illustrate the sensitivity and specificity of admission TyG and determine the optimal cut-off value for predicting BRVO. The accuracy of these predictions was quantified by calculating the areas under the ROC curves. A *p*-value of less than 0.05 was considered indicative of statistical significance.

## Results

The mean age was 61.4 ± 9.6 years in group 1 (27 women and 30 men) and 60.6 ± 10.3 years in group 2 (26 women and 24 men). The groups showed no significant differences in average age and gender distribution. (*p* = 0.707 and *p* = 0.633). No difference was found in terms of hypertension, diabetes mellitus, and cardiovascular disease (*p* = 0.599, *p* = 0.755, and *p* = 0.467, respectively). The BRVO group exhibited significantly higher triglyceride levels compared to the control group (129.8 ± 32.9 vs. 118.4 ± 20.1, *p* = 0.040). There were no statistically significant differences between the two groups in terms of low-density lipoprotein cholesterol, high-density lipoprotein cholesterol, total cholesterol, and fasting glucose levels. (*p* = 0.112, *p* = 0.119, *p* = 0.330, and *p* = 0.264, respectively). TyG index was significantly higher in group 1 (8.84 ± 0.41) when compared to group 2 (8.52 ± 0.29) (*p* < 0.001). Table 1 shows baseline characteristics, medical history details, and laboratory measurements among both groups.

**Table 1:** Patient demographic characteristics, medical history details, and baseline laboratory measurements between the groups

	Group I (n = 57)	Group II (n = 50)	p
Gender, female/male	27/30	26/24	0.633^a^
Age, years	61.4 ± 9.6	60.6 ± 10.3	0.707^b^
Hypertension (n)	21	16	0.599^a^
Diabetes mellitus (n)	8	6	0.755^a^
Cardiovascular disease (n)	7	4	0.467^a^
Glucose (mg/dL)	102.2 ± 31.1	96.4 ± 20.1	0.264^b^
Total cholesterol (mg/dL)	197.1 ± 32.8	191.2 ± 28.7	0.330^b^
Triglyceride (mg/dL)	129.8 ± 32.9	118.4 ± 20.1	**0.040**^b^
HDL-C (mg/dL)	46.1 ± 9.4	49.2 ± 10.6	0.119^b^
LDL-C (mg/dL)	127.2 ± 29.9	118.3 ± 26.9	0.112^b^
TyG index	8.84 ± 0.41	8.52 ± 0.29	** < 0.001**^b^

HDL-C: high-density lipoprotein cholesterol, LDL-C: low-density lipoprotein cholesterol, TyG index: triglyceride-glucose index

^a^ Chi-Square test

^b^Independent sample *t*-test

Logistic regression analysis was carried out to forecast the independent risk factors in discrimination between BRVO and control groups, and the TyG index (OR = 2.58; CI: 1.69–3.93; *p* < 0.001) was found to be correlated with BRVO (Table 2).

**Table 2:** Predictors of acute BRVO in multivariate regression analysis

Variable	Odds ratio (95% confidence interval)	p-value
TyG index	2.58 (1.69–3.93)	** < 0.001**
Age	0.911 (0.622–1.334)	0.776
Gender (male)	1.10 (0.750–1.602)	0.633

**Fig. 1:**
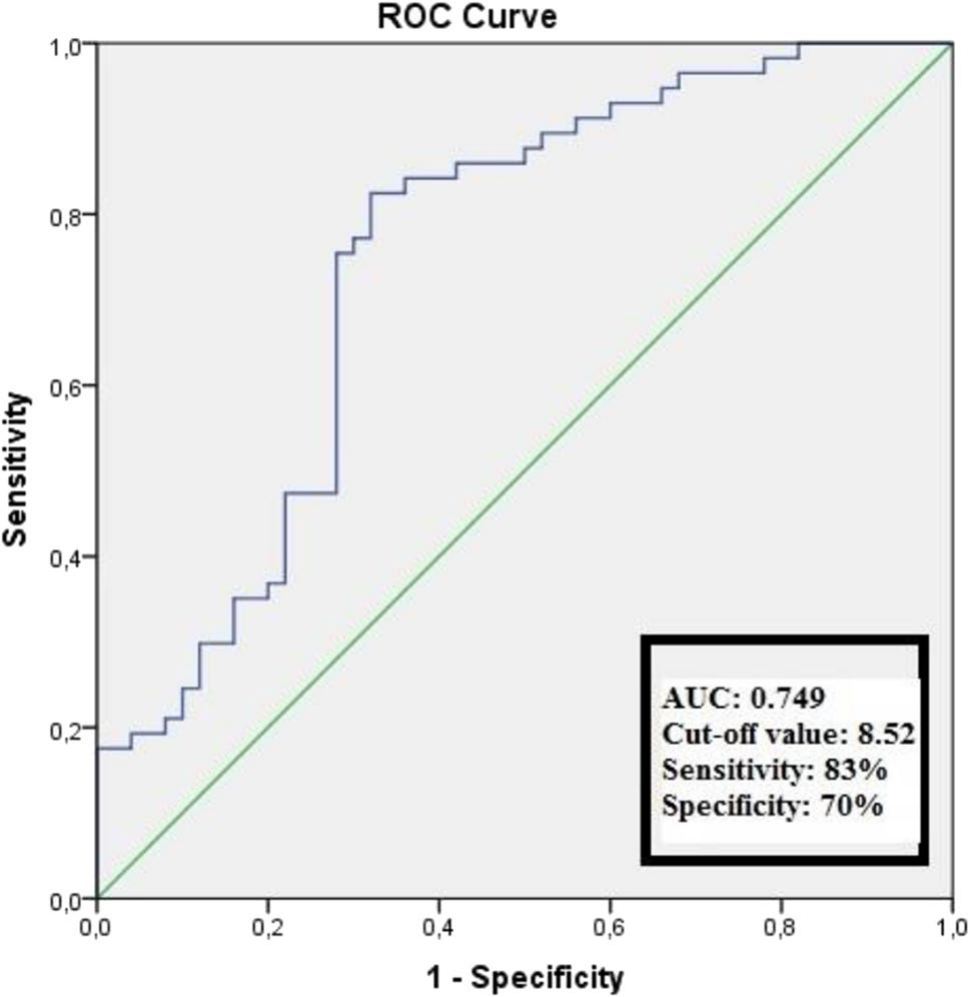
The receiver operating characteristics analysis for triglyceride‑glucose ındex in predicting branch retinal vein occlusion. AUC area under the curve, CI confidence interval

Based on the receiver operating characteristic curve, TyG exhibited an area under the curve of 0.749. The optimal cut-off value for TyG in predicting BRVO was 8.52, with 83% sensitivity and 70% specificity, as illustrated in Fig. 1.

## Discussion

In this study, we investigated TyG, a newly identified atherosclerosis biomarker in patients with branch retinal vein occlusion. Compared with healthy control participants, individuals with BRVO were found to have higher TyG; this parameter was investigated for the first time in the literature in patients with BRVO. Furthermore, based on ROC analysis, we found TyG to be a convenient index to predict disease.

Despite the great importance of BRVO in leading to sometimes severe vision loss, its etiopathogenesis remains unclear and has been the subject of some controversy. The typical occurrence of BRVOs at arterial crossings implies a potential hemodynamic disparity between arterial and venous intersections. The occluded area undergoes a pathological progression marked by degenerative alterations in the vessel walls, atherosclerosis, irregular blood constituents, and impaired blood flow. Strong associations were found between focal arteriolar narrowing and arteriovenous notching and disease occurrence [2, 12]. Arteriosclerotic changes occurring in specific areas may play a role in causing stagnation and blockage in nearby retinal veins. The risk factors linked to arterial or arteriolar diseases in BRVO could provide an explanation for its development. In cases of BRVO, the primary site of blood flow resistance consistently occurs at the AV (arteriovenous) transition point. This is typically due to vessel compression, vessel wall thickening caused by arterial disease or endothelial proliferation, or a combination of these factors [13]. A cycle of exacerbation can initiate at the occlusion site, where decreased flow leads to heightened blood viscosity, subsequently resulting in a further reduction in flow. The primary links to BRVO often stem from an elevated risk of atherosclerosis. Consequently, the primary connections to BRVO can be described as risk factors for atherosclerosis, while the remaining connections involve conditions leading to increased blood viscosity or hindered, irregular flow through retinal veins [14]. Moreover, there have been reports of numerous systemic risk factors for BRVO, including advanced age, dyslipidemia, high plasma homocysteine levels, hypertension, and diabetes, and the association of these factors with BRVO is thought to be due to atherosclerosis.

Various biomarkers have been investigated in predicting atherosclerosis, and their clinical use has been evaluated. In the past years, the TyG index, which is a valuable biomarker, has emerged as a reliable indicator of atherosclerosis [15]. In a large population and community-based sample study which includes middle-aged and elderly people, BRVO is associated with carotid artery disease, hypertension, and other cardiovascular risk factors; atherosclerosis was considered the main factor in this relationship. In a recent study, a connection was identified between elevated TyG index levels and the risk of major atherosclerosis-associated cardiac and cerebrovascular events. Another study, similar in nature, reported that an elevated TyG index might correlate with symptomatic coronary artery disease, making it a valuable adjunctive test for screening patients with heart disease. It could serve as an indicator for necessary therapeutic interventions and function as a marker of atherosclerosis [16]. Ding et al. also noted that an elevated TyG index could be linked to an increased prevalence of atherosclerotic cardiovascular diseases [17]. In this study, we found that TyG index values, acknowledged as a novel atherogenic indicator, were notably higher in the BRVO patient group compared to the control group. Furthermore, the rise in TyG levels might significantly contribute to the development of BRVO through the initiation of atherogenic events.

Another predisposing factor as well known as atherosclerosis is hypertension. In the Beaver Dam Eye Study (BDES), hypertension was associated with the prevalence of BRVO [2]. A study involving 492,488 patients over 55 years of age showed that hypertension was the main driver of increased risk for a BRVO event, with those with more severe hypertension at even greater risk [18]. The association between BRVO and hypertension is believed to stem from degenerative alterations in the vessel wall and compression of the vein at the arteriovenous crossing site [19]. In addition, hypertension plays an important role in atherosclerosis, and hardening of the retinal arteries gives rise to compression of adjacent retinal vessels within the adventitial sheath, resulting in venous stasis and making individuals more susceptible to thrombosis [20]. In recent years, numerous extensive prospective cohort studies have confirmed the strong association between the TyG index and hypertension [21, 22], and a meta-analysis determined that the TyG index is a useful predictor of the risk of developing hypertension among the general adult population [23]. Another recent study, investigating the 4-year outcomes of 19,924 individuals, revealed elevated TyG index levels in hypertensive patients, underscoring the significance of monitoring longitudinal TyG index trends for stroke prevention in individuals with hypertension, given the heightened risk of stroke and ischemic stroke associated with it [24]. Considering these studies collectively, hypertension, a significant risk factor in BRVO, substantially contributes to atherosclerosis development. The TyG index may serve as a tool to identify vessel wall degeneration and arteriovenous compression caused by hypertension. Our research indeed indicated a higher prevalence of TyG and hypertension in the BRVO group compared to the control group. TyG greater than 8.52 was found to have 70% specificity in predicting BRVO. Considering these findings and our results, we think that TyG is a good indicator of atherosclerosis and a useful parameter that can be used to predict BRVO.

While the study aimed to reduce potential sources of error, several limitations are noteworthy. Our study has limitations, including a sample size that is relatively modest, which could potentially restrict the applicability of our results to a larger population. Furthermore, our study was conducted as a cross-sectional analysis, and more robust conclusions could be drawn from longitudinal measurements of these parameters. The cross-sectional design does not allow causality inference and establishes a temporal relationship between exposure and disease; therefore, it cannot be generally determined whether the increased TyG is the root cause or is too simple a measure of other underlying causes propagated by BRVO. However, to our knowledge, this article is significant as it represents the first examination of the correlation between TyG values and BRVO.

In conclusion, the results of the study revealed that in patients with a diagnosis of BRVO in which atherosclerosis seems to play an important role, the TyG index is high and a TyG ratio value greater than 8.52 has 83% sensitivity and 70% specificity in predicting BRVO. Therefore, we suggest that high TyG level may be a useful, practical, inexpensive, and easily measurable biomarker for the assessment of BRVO development. However, further studies are needed to evaluate the role of TyG in the prognosis and treatment response of BRVO in larger samples.
